# Exploring the Motivational Drivers of Young Adults with Diabetes for Participation in Kidney Research

**DOI:** 10.1177/23743735221138236

**Published:** 2022-11-13

**Authors:** P Mohini, M Palaganas, Y Elia, L Motran, E Sochett, J Curtis, JW Scholey, L McArthur, FH Mahmud

**Affiliations:** 1 7979 Division of Endocrinology, Department of Pediatrics, The Hospital for Sick Children, Toronto, Canada; 2 7989 Division of Nephrology, Department of Medicine, University Health Network, Toronto, Canada; 3 7938 Institute of Medical Sciences, University of Toronto, Toronto, Canada

**Keywords:** motivational drivers, barriers, diabetes, research, patient engagement

## Abstract

Understanding motivational drivers and barriers to patient participation in diabetes research are important to ensure research is relevant and valuable. Young adults with type 1 diabetes (T1D) completed a 31-question qualitative survey evaluating participant experience, understanding, and motivators and barriers to research involvement. A total of 35 participants, 19–28 years of age, 60% female, completed the survey. Motivating factors included personal benefit, relationship with the study team, curiosity, financial compensation, altruism, and nostalgia. Older participants (>22 years) reported higher levels of trust in the study team (p = 0.02) and their relationship with the study team positively influenced their decision to participate (p = 0.03). Financial compensation was a strong motivator for participants with higher education (p = 0.02). Age, sex, education level, and trust in the study team influenced participants’ understanding. Barriers included logistics and lack of familial support. Important motivational drivers and barriers to participation in research by young adults with T1D must be considered to increase research engagement and facilitate the discovery of new knowledge.

## Introduction

Engaging young adults with type 1 diabetes (T1D) in the co-design of diabetes research has the potential to advance care and improve self-management (1). Even so, relatively little is known about what motivates young adults with T1D to participate in clinical research (2–4), while in other patient populations, various motivational drivers and barriers have been shown to influence a patient's decision to participate (5–10).

Other patient populations found health benefits, having access to health care providers, services, and treatment, as well as altruism to be commonly reported motivators to participation in research (6), while lack of trust and risks associated with protocol procedures were commonly reported barriers (8). Other barriers including inconvenience, not enough compensation, and the possibility to receive placebo were also reported, however, less common (6,8). Illness and severity of disease (5), and health inequities including gender, race, ethnicity, and social determinants of health (11–14), were also shown to influence the participation of other populations in research. Similarly, retention practices including, study information, study visit characteristics, reminders and flexibility, benefits versus risks, and financial incentives are shown to reduce attrition rates, positively influencing a patient's continued motivation to participate in clinical research (15,16).

Addressing this gap in knowledge in a population of patients with T1D, is only becoming more important as the incidence of T1D and its complications, in particular kidney disease, continues to increase (17,18). The Canadians Seeking Solutions and Innovations to Overcome Chronic Kidney Disease (Can-SOLVE CKD) is a national patient-oriented kidney research network in Canada, that focuses on patient priorities and patient engagement in the research process (19). As part of this network, the *Early Determinants of Cardio-Renal Disease in Youth with Type 1 Diabetes Study* that is evaluating different strategies on how to prevent and/or delay diabetes complications in young adults with T1D, provided the opportunity to explore why persons with diabetes engage in research. The objective of this study was to therefore understand the motivational drivers and barriers of young adults with T1D for engaging in clinical research.

## Methods

### Early Determinants of Cardio-Renal Disease in Youth with Type 1 Diabetes Study (“Study”)

The *Early Determinants of Cardio-Renal Disease in Youth with Type 1 Diabetes Study* (“Study”) is a longitudinal, observational study aimed at (1) identifying who is at risk for progressive decline in kidney function, (2) predicting a decline in kidney function using biological and cardiovascular markers, and lastly (3) looking at how psychosocial, and social factors modify risk for kidney disease in young patients with T1D. Secondary to this overarching study aim, we also explored using qualitative methods why young people with diabetes participate in this research, and in particular why they agreed to testing of their kidney function.

#### Kidney Function Test

In this study, kidney function was measured using Measured Glomerular Filtration Rate (“Kidney Function Test”; 20). This Kidney Function Test is a more precise measure of early changes in kidney function, which may be a helpful early indicator of diabetic kidney disease. It is an involved test requiring intravenous lines for infusion of iohexol, a contrast agent used for X-ray imaging, and frequent blood draws, and is a very lengthy procedure (i.e., approx. 6 h) so not very practical for patients as well as clinics and clinicians. Due to the nature of this test, it is not widely available in a clinical setting and is mostly used for research purposes. The kidney function test was offered as an optional test in this study. At the time of consent, potential participants were informed about the benefits of participating in the Study and the Kidney Function Test, receipt of individualized Kidney Function Test results at the completion of the Study, and receipt of monetary compensation and reimbursement to cover their time and any expenses incurred during their participation, respectively.

### Patient Population

Young adults with T1D between the age of 19 to 28 years who completed the Kidney Function Test as part of the Study at the Hospital for Sick Children in Toronto, Canada and who agreed to participate in exploring the motivational factors and barriers for their participation in this test were included in this study. Patient demographics collected and included in this study were age, sex, education level, and occupation status.

### Qualitative Study Survey (“Survey”)

Patients’ perspectives were collected using a qualitative study Survey (“Survey”). The Survey used in this study was derived from a validated 41-item based questionnaire designed by Arnetz et al (21) containing six patient perception domains including motivation, risks and benefits of participation, the nature of the study itself, and practical considerations such as cost and convenience. This 41-item questionnaire is in pilot format requiring testing in larger and more diverse populations but was nonetheless the tool of choice for this exploratory qualitative Study since it was developed for the intended use with any patient group, regardless of diagnosis.

Adaptation of this questionnaire involved collaboration between our research group and patient partner(s) to be able to achieve a Survey that was specific to our patient cohort and Study. The adapted version of the 41-item questionnaire was distilled down to a 31-item Survey focused on measuring the patient's (1) study experience, (2) understanding of why they are participating in this research (i.e., why the Kidney Function Test was conducted, potential risks associated with the test), (3) relationship with the Study team, (4) perceived benefits of participation, and (5) potential motivating factors and barriers influencing their participation. The Survey also collected the patient's demographic information described earlier.

All survey responses were anonymously collected using a RedCAP platform. See Appendix for adapted 31-Question Survey.

### Statistical Analysis

The Survey employed a combination of a 4-point Likert scale (i.e., completely agree—1, somewhat agree—2, somewhat disagree—3, and completely disagree—4) to determine the level of agreement to specific and open-ended short answer questions regarding Study experience, curiosity toward the test, relationship with the Study team, financial compensation, and personal benefit from participation. A neutral option was not provided.

A Spearman Rank Correlation test was performed on questions that used the Likert scale to determine the relationship(s) between the variables and Spearman's Rank Correlation coefficient or Spearman's ρ were included where relevant. The test was also performed on four demographic subsets (male, female, ≦22 [19–22 years of age], and >22 [23–28 years of age]) to investigate the influence of sex and age on the relationships between the variables. Twenty-two years of age was selected as the cut-off for comparison as it is the median age for completion of a bachelor’s degree and described as an age during which the transition to young adulthood may occur (22). As an explorative study, we were interested in the views of participants descriptively. A p-value of <0.05 was considered statistically significant and reported in the results section and is provided for additional detail, recognizing the limited power to detect differences in this sample.

Open-ended survey questions were thematically analyzed, and no statistical analysis was performed on these responses.

## Results

### Demographics

Ninety-four young adults were approached to complete a Kidney Function Test as part of the Study. Of these, 53 consented, while 41 did not consent due to scheduling conflicts [23], ineligibility [9], and COVID-19 [2]. Of the remaining participants, seven were lost to follow-up. Of the 53 patients that participated in the Kidney Function Test, 35 completed the Survey; 14 males (40%) and 21 females (60%) aged 19 to 28 years. Patient demographics are shown in Table 1.

**Table 1.** Patient Demographics.

Variable	Level	n (%)
Age	≦22	17 (49)
	>22	18 (51)
Sex	Male	14 (40)
	Female	21 (60)
Education Level Reached	High school	10 (29)
	Trade school	0 (0)
	Undergraduate—College	0 (0)
	Undergraduate—University	15 (43)
	Graduate—College	5 (14)
	Graduate—University	4 (11)
	Post-graduate	1 (3)
Occupation at the time of participation	Working, full time	10 (29)
	Working, part-time	4 (11)
	Unemployed	2 (6)
	Student, full time	18 (51)
	Student, part-time	1 (3)
	Homemaker/on leave	0 (0)

### Factors Influencing Participation

**Figure 1.**
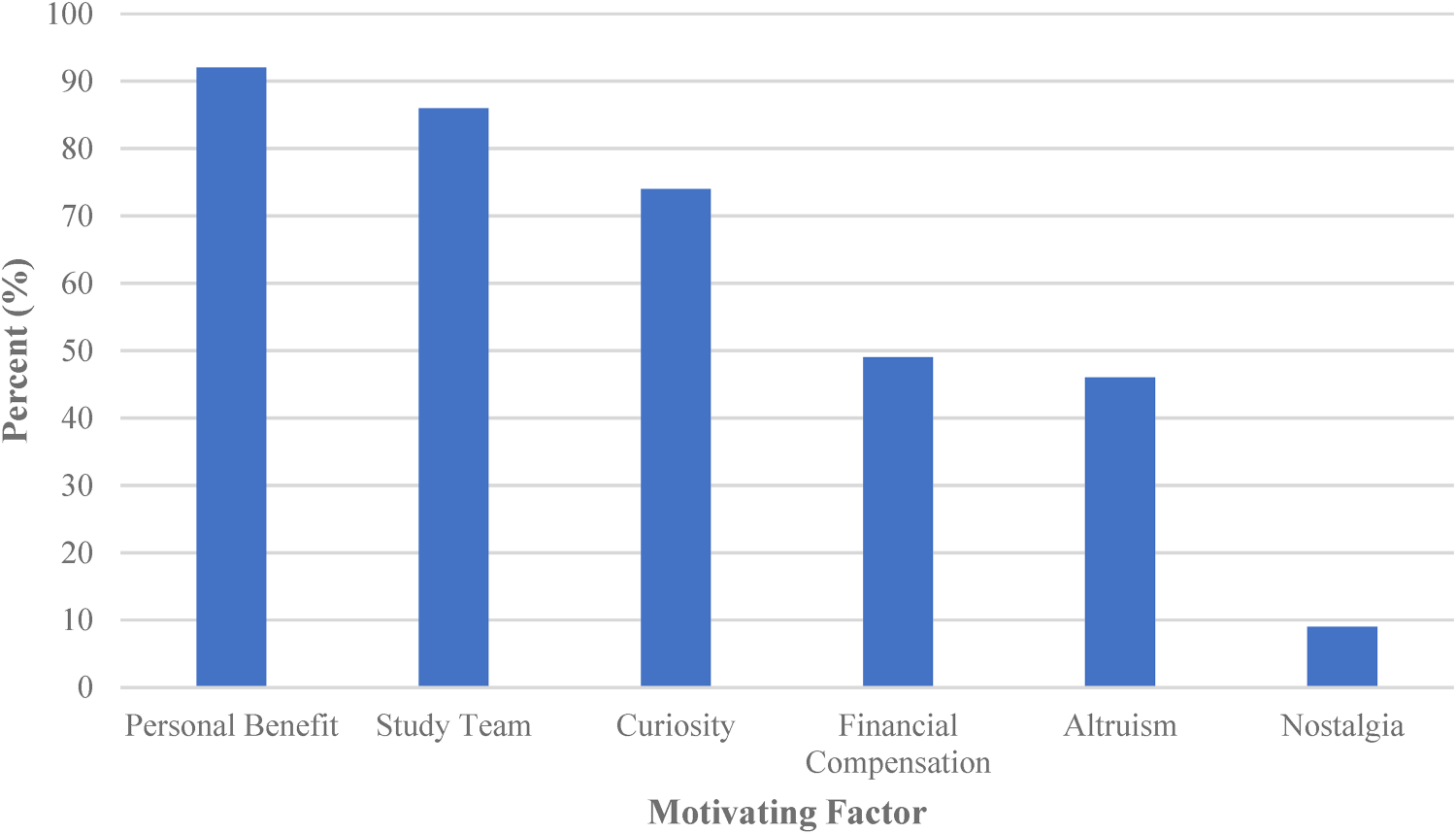
Summary of factors influencing participation.

Personal benefit was the most common factor influencing participation with 92% of participants indicating that they benefitted from participating in the Kidney Function Test (Figure 1). Perceived benefits included access to a test that would not be available to them under standard clinical care,an opportunity to have their kidney function monitored, learning more about diabetes, obtaining their personal results, and receiving financial compensation.

A substantial portion of participants surveyed, 86%, felt that their relationship with the Study team strongly influenced their decision to participate in the Kidney Function Test (Figure 1). All participants reported that they trusted the Study team and felt comfortable and respected. Older participants had a higher level of trust in the Study team (ρ = 0.02) and indicated that their relationship with the Study team had a greater, positive effect on their decision to participate (ρ = 0.03). No correlation was seen between the participants’ level of trust in the Study team and the care with which they read the consent form for the entire cohort (ρ = 0.03). A weak correlation was seen between the level of trust in the Study team and participants understanding of why the Kidney Function Test was being done (ρ = 0.19).

Curiosity was the third strongest motivating factor, reported by 74% of those surveyed (Figure 1). From this group, 43% were curious about their personal results, 23% about the procedure itself, 20% about the impact of diabetes on their health, and 9% about the impact of the Study findings on the care of people with T1D in the future.

Financial compensation was an influential factor for 49% of the participants (Figure 1). Those with a higher level of education were more likely to indicate that financial compensation had a greater influence on their decision to participate (ρ = 0.02). Participants influenced by financial compensation and those who had to miss school or work were less satisfied with the amount of financial compensation provided. Participants who were students at the time of participation tended to be more satisfied with the financial compensation than the participants who were working.

Altruism was a motivating factor for 46% of the Survey participants, who expressed that helping advance diabetes research and improve the lived experience for future persons with diabetes were the reasons for their participation (Figure 1).

A smaller portion of patients (9%) indicated they were motivated by nostalgia (i.e., sentimental connection to the staff and care received at the Hospital for Sick Children; Figure 1). They reported that Study participation allowed them to come back to The Hospital for Sick Children again and feel like a SickKids patient for the day.

#### Impact of sex on motivating factors

**Figure 2.**
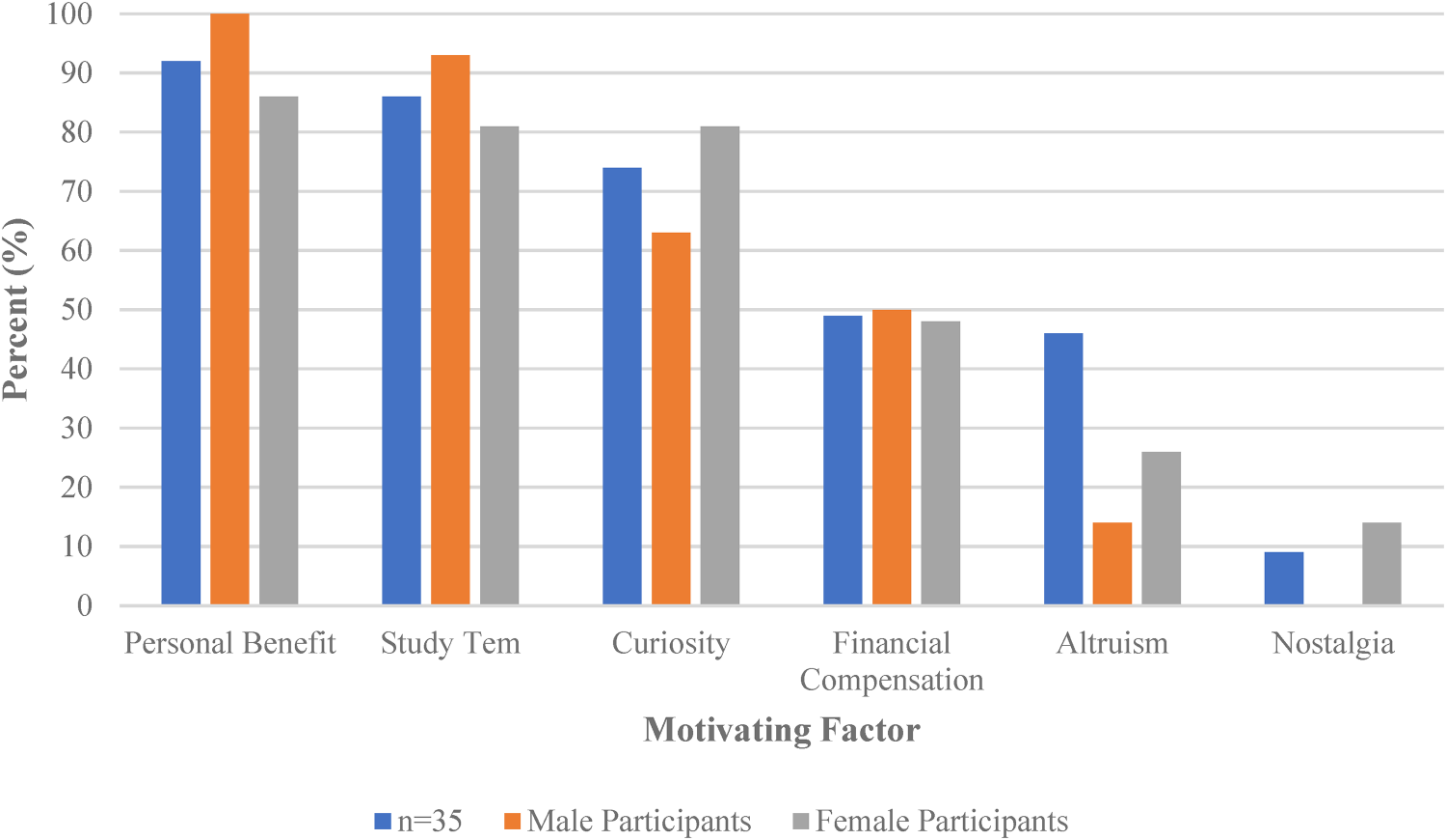
Summary of the impact of sex on motivating factors.

Males reported personal benefit as a motivating factor more often (100%) than females (86%; Figure 2). In addition, females felt that they benefitted more from the Study than males. The Study team was an important motivating factor for participation for 93% of the male participants and 81% of the female participants (Figure 2). Among Survey participants who indicated high levels of trust in the Study team, males tended to report reading the consent form more carefully, whereas female participants tended to read the consent form less carefully. The Spearman Rank Correlation showed that for male participants, relationship with the Study team affected their decision to participate, and the greater the relationship the less they understood why the Kidney Function Test was being done. For female participants, their relationship with the Study team affected their decision to participate, and their relationship was proportional to their understanding of why the Kidney Function Test was being done. Differences in other motivational drivers emerged: curiosity (81% of females vs 63% of males), altruism (26% of females vs 14% of males), and nostalgia (14% of females and 0% of males; Figure 2).

#### Impact of age on motivating factors

**Figure 3.**
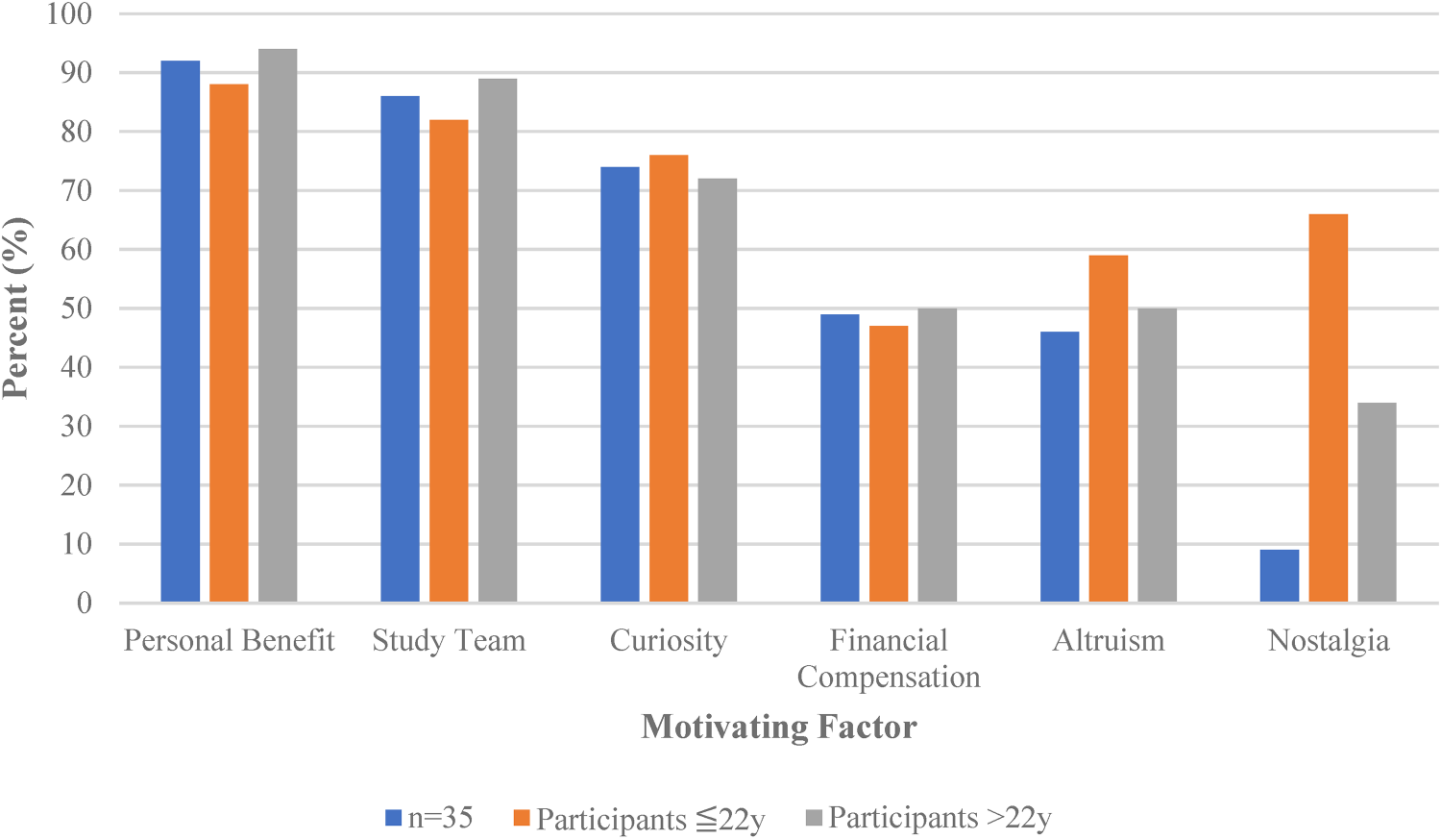
Summary of the impact of age on motivating factors.

Trends were observed across age groups (19–22 and 23–28 years of age) (Figure 3). Participants above the age of 22 years were more likely to cite personal benefit (94% vs 88%) and their relationship with the Study team (89% vs 82%) as motivating factors to participation in this Study (Figure 3). For the older group, there was a negative correlation between the Study team as a motivating factor and their level of understanding about the purpose of the Kidney Function Test. Younger patients were more likely to report altruism (59% vs 50%) and nostalgia (67% vs 33%) as motivating factors (Figure 3).

No correlation was seen between age or level of education and curiosity toward the Study. There was no relationship between age and financial compensation as an incentive to participate. Older participants felt they were more fairly compensated for their time and that they benefitted from participating in the Study.

### Barriers to Participation

Seven participants (20%) faced barriers when participating in the Study. Five said that they lacked familial support in participating, and four said that they faced challenges getting to SickKids, including a long commute (i.e., ∼100 km) and bad weather, or difficulty arranging a Study visit around their work schedule.

### Reported Experience

Overall, 54% of the participants reported a positive Study experience. Through open-ended responses, participants stated that the Study team was nice and friendly and that the long Kidney Function Test gave them time to relax and catch up on movies and sleep. Some participants (31%) reported discomfort from the Kidney Function Test since the procedure was long, invasive, timed urine collection inconvient and required multiple intravenous catheters without opportunity for a break. 

### What Participants Want to Know

When participants were asked using open-ended questions what they were interested in wanting to know about their participation in the Study and Kidney Function Test, they stated that they were interested in their personal results (i.e., Kidney Function Test, and other tests completed as part of the overarching Study including the bone scan, and blood work), the overall Study results, and the impact the Study would have on clinical care and research advancement. Participants wanted to know the average results, results broken down by age groups, and how the results differ from non-diabetic individuals.

## Discussion

Our Survey identified six key factors that influence patient participation in clinical research including personal benefit, relationship with the Study team, curiosity, financial compensation, altruism, and nostalgia. Personal benefit (23–28), relationship with the Study team (29,30), financial compensation (25,27,29–32), and altruism (23,31,33,34) as motivational drivers to participate in clinical research are consistent with the existing literature in populations with other diseases, while nostalgia has not been described in the extant literature and as such a unique finding in this Study.

Personal benefit and relationship with the Study team were greater motivational drivers for older participants >22 years of age and interestingly for males. While we have no explanation for this trend among the male participants, it is possible that older patients may be more cautious and proactive about preventing and delaying diabetes-related complications. In addition, older patients have had a longer association with the institution and Study team and have had more time to build strong relationships. The correlation between trust in the Study team and willingness to participate in research has been previously described (23,29,34–36) however, we believe that the relationship between trust and understanding the purpose of the Study, as well as the relationship between trust and the care with which participants read the consent form, are novel findings of the Survey. In the context of type 1 diabetes, the pediatric care team has a deep and long-standing relationship with their patients and has provided medical and emotional support spanning initial diagnosis through adolescence. It has been reported that 64% of young adults avoided transitioning to adult care due to the strong emotional bond with their pediatric teams (37), and this positive attachment likely extends into clinical research.

There was no correlation between financial compensation influencing participation, and the participant's age and occupation. Financial compensation was a greater motivating factor for participants who had reached a higher level of education, and this may reflect the reality of balancing the opportunity cost to participating in the study and other competing priorities. Financial compensation would then serve as an important additional incentive.

Altruism based on a desire to help the diabetes community and advance diabetes research was also important factors for participants. Two participants explicitly stated that they did not enjoy their experience but nevertheless participated to help with the research.

Nostalgia, a unique finding in this Study, was reported as a motivational driver to participation in research twice as many times by female participants ≤22 years of age in this Study. Nostalgia is known to increase a person's state of openness to engaging in experience(s) (38), and this openness tends to rise during young adulthood (39,40). Additionally, no significant gender differences in nostalgia have been reported literature (39). It is well established in the diabetes literature that many persons with diabetes struggle with transition from pediatric to adults care (41) with worsening of diabetes control and treatment satisfaction (42). One report described 38% of patients were less satisfied with their adult care (42). As such, the nostalgia experienced by the young adult female participants in this Study, where those individuals experienced positive feelings about their previous participation in research with the Study Team, likely were encouraged by the state of openness to participate in other clinical research including the Kidney Function Test.

A lack of familial support was found to be the primary barrier to participation in this Study. This factor has not been identified in other studies. We believe that this barrier may be more important for young adults and requires further investigation.

## Limitations

One of the limitations of this Study was use of an online Survey for data collection. Given the impact of COVID-19 on in person conduct of clinical research, this was the only feasible way to administer the Survey to participants. This method, however, may have limited the scope of participant responses to the Survey, where the research team would miss more nuanced responses to the survey questions. The open-ended survey questions may have also been understood and interpreted differently by the participants. Another limitation of this Study is that the participants were already involved in the overarching longitudinal research study and therefore the findings of the Survey may not be generalizable to de novo populations of young adults with T1D. Additionally, the individuals that declined to participate in the Kidney Function Test were not approached to better understand the barriers to their participation because the the ethics approval only allowed for survey of consented individuals. This information, however, could be helpful to increase recruitment and participation in other diabetes research studies. Lastly, the research teamrecognizes that an absolute age to describe the emergence of adulthood shows variability and may not apply to every individual, but this allowed for additional evaluation of the information collected.

## Conclusion

Identifying the motivational drivers and barriers young adults with T1D face when participating in clinical research, is important to help researchers design future studies that will increase recruitment and retention rates. By doing so, this ensures research is relevant and valuable for eventual implementation into clinical care for people with diabetes. We identified a number of motivators and barriers, some of which have been found in other populations and one that is unique to our patient population, but more research is required to be able to understand these motivators and barriers in this population and how they can effectively engage young adults with T1D in clinical research studies.

## Appendix

### Kidney Function Test—Patient Participation Questionnaire

The AdDIT/SPOR study team would like to better understand the factors that made you agree to participate in the optional Kidney Function Test. Through this questionnaire, the study team hopes to learn from your experience to be able to improve research studies. This questionnaire will take approximately 15 min to complete.

**Table:**

The first set of questions are about Kidney Function Test				
1. You received clear information about the test itself when you were approached about participating in the Kidney Function Test.	□ Agree Completely	□ Agree Somewhat	□ Disagree Somewhat	□ Disagree Completely
2. You received clear information about the length of the test when you were approached about participating in the Kidney Function Test.	□ Agree Completely	□ Agree Somewhat	□ Disagree Somewhat	□ Disagree Completely
3. You received clear information about what would be asked from you when you were approached about participating in the Kidney Function Test.	□ Agree Completely	□ Agree Somewhat	□ Disagree Somewhat	□ Disagree Completely
4. You read the participation agreement when you were agreeing to participate.	□ Agree Completely	□ Agree Somewhat	□ Disagree Somewhat	□ Disagree Completely
5. You were able to comfortably share your views and ask questions regarding the Kidney Function Test with the study team?	□ Agree Completely	□ Agree Somewhat	□ Disagree Somewhat	□ Disagree Completely
a. Your views were respected, and your questions were properly answered by the study team.	□ Agree Somewhat		□ Disagree Somewhat	□ N/A
	□ Agree Completely		□ Disagree Completely	
6. You trusted the study team.	□ Agree Completely	□ Agree Somewhat	□ Disagree Somewhat	□ Disagree Completely
7. Your relationship/comfort level with the doctor(s), nurse(s) and/or research coordinator(s) affected your decision to participate.	□ Agree Completely	□ Agree Somewhat	□ Disagree Somewhat	□ Disagree Completely
8. You understood why the Kidney Function Test was being done before you consented to participate.	□ Agree Completely	□ Agree Somewhat	□ Disagree Somewhat	□ Disagree Completely
9. You fully understood the risks associated with the Kidney Function Test before you consented to participate.	□ Agree Completely	□ Agree Somewhat	□ Disagree Somewhat	□ Disagree Completely
10. If you disagreed to either of the past two questions, please mention why you still consented to participate.				
11. You were curious about/interested in the Kidney Function Test at the time you agreed to participate.	□ Agree Completely	□ Agree Somewhat	□ Disagree Somewhat	□ Disagree Completely
12. If you answered “Yes” to the previous question, please mention what you were curious about.				
13. You were fairly compensated for your time.	□ Agree Completely	□ Agree Somewhat	□ Disagree Somewhat	□ Disagree Completely
14. The monetary compensation influenced your decision to participate.	□ Agree Completely	□ Agree Somewhat	□ Disagree Somewhat	□ Disagree Completely
15. You bore out-of-pocket costs to participate in the Kidney Function Test.	□ Yes Approx. $______		□ No	
16. You had to miss school/work to participate in the Kidney Function Test.	□ Yes		□ No	
17. You feel you personally benefitted from participating in the Kidney Function Test.	□ Agree Completely	□ Agree Somewhat	□ Disagree Somewhat	□ Disagree Completely
18. You feel your participation in the Kidney Function Test will help others in the future.	□ Agree Completely	□ Agree Somewhat	□ Disagree Somewhat	□ Disagree Completely
19. You had familial support to participate in the Kidney Function Test.	□ Agree Completely	□ Agree Somewhat	□ Disagree Somewhat	□ Disagree Completely
20. Please describe your experience from the Kidney Function Test.				
21. Were there other factors that motivated you to participate in the Kidney Function Test despite it being long and a bit uncomfortable? If Yes, please indicate below				
22. Do you think something could have been done to make the Kidney Function Test more comfortable?				
23. What would you like to know about the results from this study?				
24. You faced challenges getting to SickKids.	□ Agree Completely	□ Agree Somewhat	□ Disagree Somewhat	□ Disagree Completely
25. If you agreed to the previous question, please elaborate.				
**Before we end, we would like to know a bit more about you.**				
26. What is the highest level of education you have reached?	□ High School □ Trade School □ Undergraduate—College □ Undergraduate—University □ Graduate—College □ Graduate—University			
27. What was your occupation at the time of participation? Please select all that apply.	□ Working full time □ Working part time □ Unemployed □ Student—Part Time □ Student—Full Time □ Homemaker/On leave			
28. Apart from AdDIT and SPOR, have you participated in any other research studies?	□ Yes		□ No	
29. Do you feel like you personally benefitted from AdDIT and SPOR?	□ Yes		□ No	
30. If you answered “Yes” to the previous question, please elaborate.				
31. Is there anything else you would like the study team to know?				
Thank you for completing this questionnaire!
